# The Mystery of Episodic Recurrent Jaundice in a Young Male: Cholestasis With a Normal Gamma-Glutamyl Transferase

**DOI:** 10.7759/cureus.13834

**Published:** 2021-03-11

**Authors:** Samiksha Gupta, Ijlal Akbar Ali, Eleanor Abreo, Veena Gujju, Maham Hayat

**Affiliations:** 1 Internal Medicine, University of Oklahoma Health Sciences Center, Oklahoma City, USA; 2 Digestive Diseases and Nutrition, University of Oklahoma Health Sciences Center, Oklahoma City, USA; 3 Pathology, University of Oklahoma Health Sciences Center, Oklahoma City, USA

**Keywords:** benign recurrent intrahepatic cholestasis, rare liver disease, benign recurrent intrahepatic cholestasis (bric), normal gamma-glutamyl transferase (ggt)

## Abstract

Benign recurrent intrahepatic cholestasis (BRIC) is a very rare autosomal recessive genetic disorder which presents with recurrent jaundice. We report the case of a young male with a history of methamphetamine use who presented with recurrent episodes of right upper quadrant abdominal pain, vomiting, dark urine, and pale stools. These symptoms always resolved within four weeks of presentation. During these episodes, the patient had a cholestatic pattern derangement of liver function tests with a normal gamma-glutamyl transferase (GGT). Workup for abnormal transaminases was unremarkable. A percutaneous liver biopsy obtained on the third visit was notable for a parenchymal lobule that exhibited slight Kupffer cell hyperplasia and subtle evidence of canalicular cholestasis. There was no evidence of cirrhosis, steatosis, hepatitis, or malignancy. Thus, a diagnosis of BRIC was made, and the patient was managed conservatively. Recognition of this rare entity is critical since its benign natural history is reassuring for the patient, and physicians can refrain from repetitive expansive and costly workups.

## Introduction

Benign recurrent intrahepatic cholestasis (BRIC), also known as Summerskill-Walshe-Tygstrup syndrome, is an autosomal recessive genetic disorder characterized by recurrent intrahepatic cholestasis which does not progress to cirrhosis or end-stage liver disease. It is one phenotypic end of a spectrum of disorders caused by mutations in genes that affect the excretion of bile into the canaliculi and thus causes stasis of bile salts. Only a few hundred cases have been reported in the literature since it was first described by Summerskill and Walshe in 1959 [1]. Most reported cases have occurred in Asian countries, such as Japan, China, and India [2]. We herein report a unique case of BRIC from the United States. 

## Case presentation

A 26-year-old lean Caucasian male presented with recurrent episodes of right upper quadrant abdominal pain, vomiting, dark urine, pale stools, and jaundice. His first episode occurred at the age of 22, followed by a second episode at age 23 and a third at age 24. The patient endorsed frequent pruritus. He denied fevers, chills, weight changes, or altered bowel habits. He adamantly denied ever taking any over-the-counter medications, herbal supplements, steroid injections, or alcohol use.

Physical examination revealed scleral icterus and scratch marks all over his body. Investigations during each visit showed abnormal liver function tests: total bilirubin 33.1 mg/dL (predominantly conjugated), aspartate aminotransferase (AST) 39 U/L, alanine transaminase (ALT) 57 U/L, alkaline phosphatase (ALP) 289 U/L, and gamma-glutamyl transferase (GGT) 15 U/L. Ultrasound and CT scan of the abdomen were unremarkable. A ferritin level was normal, and C282Y and H63D mutations were not detected. Workup for viral and autoimmune hepatitis, alpha-anti trypsin deficiency, viral serology, and Wilson’s disease was negative.

The patient’s symptoms improved spontaneously within four weeks of each episode. A percutaneous liver biopsy obtained on the third visit was notable for a parenchymal lobule that exhibited slight Kupffer cell hyperplasia and subtle evidence of canalicular cholestasis.

**Figure 1:**
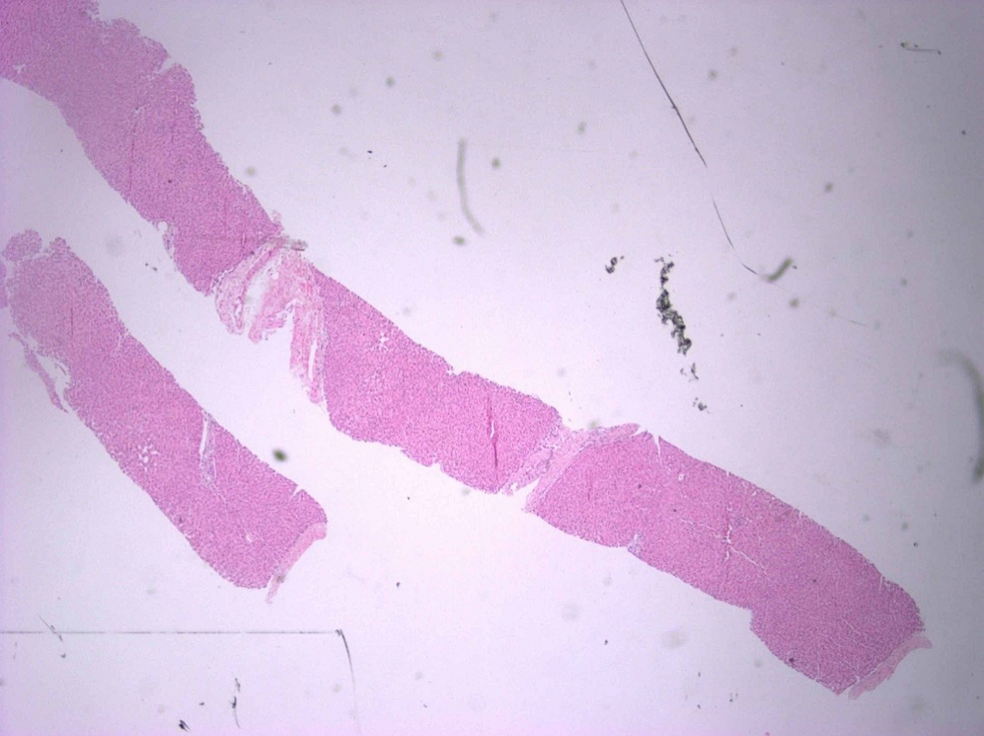
Hematoxylin and eosin-stained liver biopsy at 4x magnification The initial impression at the low-power view is of a benign liver with preserved lobular architecture and preserved anatomical relationships between the portal tracts and central veins. Notably, the biopsy is lacking signs of cirrhosis, steatosis, and hepatitis.

**Figure 2:**
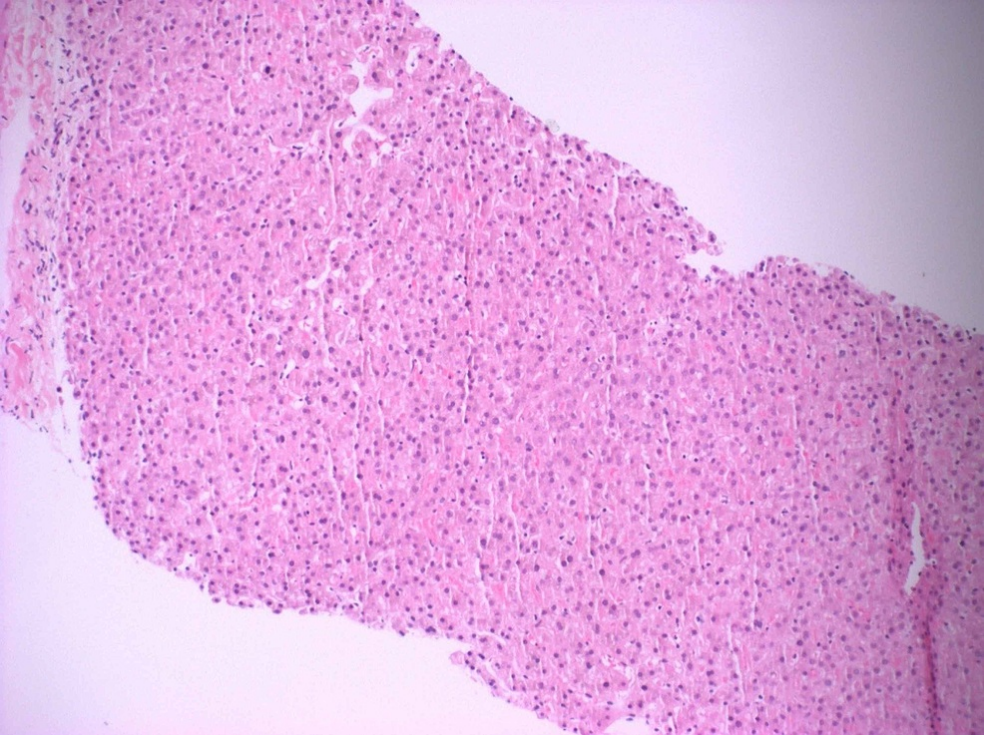
Hematoxylin and eosin-stained liver biopsy at 10x magnification This high-power image demonstrates subtle Kupffer cell hyperplasia. This view confirms the negative findings initially noted at low-power

There was no evidence of cirrhosis, steatosis, hepatitis, or malignancy. He had undergone nearly three rounds of imaging studies and serological tests at different hospitals before a diagnosis of BRIC was confirmed on liver biopsy (Figures 1-2). He was managed conservatively with ursodeoxycholic acid and had symptomatic relief with dramatic improvement in the total bilirubin to 1.6 mg/dL (baseline) from 33.1 mg/dL. He was discharged to follow-up in the gastroenterology clinic. He has since had a few more episodes which have been managed as an outpatient.

## Discussion

Genetic intrahepatic cholestasis disorders include progressive familial intrahepatic cholestasis (PFIC), BRIC, intrahepatic cholestasis in pregnancy, and drug-induced intrahepatic cholestasis, including cholestasis induced by hormonal contraceptives [3]. The disease course of BRIC is benign without progression to fibrosis or end-stage liver disease (ESLD). PFIC is a progressive variant of BRIC. Affected individuals typically present in early infancy with jaundice, severe pruritis, hepatosplenomegaly, and growth retardation with progression to ESLD and death in the first or second decade of life [4].

There are two major genes that affect the excretion of bile acids. The first gene, ATP8B1, is located on chromosome 18q and encodes for a P-type ATPase. Mutations in this gene decrease the stability of the canalicular membrane and function of transmembrane transporters, which results in BRIC type 1. The second gene is ABCB11 on chromosome 2q31. Mutations in ABCB11 disturb the function of the bile salt export pump (BSEP), which is the primary transporter of bile acids from the hepatocyte to the biliary system. These mutations result in BRIC type 2 [5-6].

The precipitating factors for cholestasis in BRIC are not well-defined. A review of the literature showed that episodes have been reported following infection, fever, and certain medications, such as oral contraceptive pills [7]. The diagnostic criteria of BRIC include at least two episodes of jaundice and pruritis lasting weeks to months. Episodes are separated by a symptom-free interval lasting anywhere from several months to years [8]. Patients typically present during the first two decades of life. There may be a history of steatorrhea and weight loss because of malabsorption due to loss of fat-soluble vitamins. Associated features also vary with the subtype of BRIC. Patients with BRIC Type 1 often have hearing loss, pancreatitis, and diarrhea, whereas patients with BRIC Type 2 have cholelithiasis and a higher risk of hepatobiliary malignancy early in life. Physical examination typically reveals icterus, excoriations due to itching, and/or hepatomegaly. Liver function test abnormalities typically present a few weeks after the development of pruritus. Alkaline phosphatase elevations occur first, followed by serum bilirubin elevations (primarily conjugated). Aminotransferases are typically normal or mildly elevated. A normal or mildly elevated GGT level is classic for BRIC. Globulin levels are not altered which differentiates it from other diseases like autoimmune hepatitis and primary biliary cirrhosis. Liver biopsy is useful in distinguishing BRIC from other conditions characterized by episodic cholestasis. Centrilobular cholestasis on liver biopsy is pathognomonic for BRIC. Less specific biopsy findings include pericentral hepatocellular degeneration, hepatocyte necrosis and inflammation in areas lacking bile pigment, focal lobular mononuclear cell infiltrate, portal inflammation, and cholangiogram proliferation [2]. Molecular testing can help confirm the diagnosis and differentiate subtypes to help study mutation-targeted therapeutic strategies but is not required to establish the diagnosis [9].

There is no cure for BRIC disease, and management consists of symptomatic relief only. The first-line treatment for cholestatic episodes in BRIC patients is oral ursodeoxycholic acid (UDCA) which works to enhance the hepatobiliary secretion of bile salts. Other medications that have been successfully tried, including rifampicin which upregulates export pumps and cholestyramine which binds bile salts and prevents re-absorption in the enterohepatic circulation [5, 9]. Yakar et. al reported a series of 16 patients refractory to medical therapy who had temporary nasobiliary drains placed with resultant improvement in liver enzymes and pruritis within three days of the procedure [10]. Plasmapheresis and extracorporeal liver support therapy have also been used successfully in a few cases of BRIC and helped resolve the cholestasis episodes [11].

## Conclusions

BRIC is a very rare benign cause of cholestasis that is important to keep in the differential when working up patients with recurrent cholestasis episodes. A normal GGT and specific findings on liver biopsy help establish the diagnosis. Recognition of this rare entity is critical since its benign natural history is reassuring for the patient, and physicians can refrain from repetitive expansive and costly workups.

## References

[1] Schapiro RH, Isselbacher KJ (1963). Benign recurrent intrahepatic cholestasis. N Engl J Med.

[2] Luketic VA, Shiffman ML (2004). Benign recurrent intrahepatic cholestasis. Clin Liver Dis.

[3] Sticova E, Jirsa M, Pawłowska J (2018). New insights in genetic cholestasis: from molecular mechanisms to clinical implications. Can J Gastroenterol Hepatol.

[4] Knisely AS, Bull LN, Shneider BL (2014). ATP8B1 Deficiency. GeneReviews® (Internet).

[5] van der Woerd WL, van Mil SW, Stapelbroek JM, Klomp LW, van de Graaf SF, Houwen RH (2010). Familial cholestasis: progressive familial intrahepatic cholestasis, benign recurrent intrahepatic cholestasis and intrahepatic cholestasis of pregnancy. Best Pract Res Clin Gastroenterol.

[6] Soroka CJ, Boyer JL (2014). Biosynthesis and trafficking of the bile salt export pump, BSEP: therapeutic implications of BSEP mutations. Mol Aspects Med.

[7] Reichert MC, Hall RA, Krawczyk M, Lammert F (2018). Genetic determinants of cholangiopathies: molecular and systems genetics. Biochim Biophys Acta Mol Basis Dis.

[8] Schonfeld EA, Brown RS Jr (2017). Genetic testing in liver disease: what to order, in whom, and when. Clin Liver Dis.

[9] van der Woerd WL, Houwen RH, van de Graaf SF (2017). Current and future therapies for inherited cholestatic liver diseases. World J Gastroenterol.

[10] Yakar T, Demir M, Gokturk HS, Unler Kanat AG, Parlakgumus A, Ozer B, Serin E (2016). Nasobiliary drainage for benign recurrent intrahepatic cholestasis in patients refractory to standard therapy. Clin Invest Med.

[11] Saich R, Collins P, Ala A, Standish R, Hodgson H (2005). Benign recurrent intrahepatic cholestasis with secondary renal impairment treated with extracorporeal albumin dialysis. Eur J Gastroenterol Hepatol.

